# An Absolute Light Scattering Photometer: II. Direct Determination of Scattered Light From Solutions

**DOI:** 10.6028/jres.068A.007

**Published:** 1964-02-01

**Authors:** Donald McIntyre

## Abstract

The light scattering photometer recently described in this journal by McIntyre and Doderer has been examined to determine its ability to measure the absolute scattering of liquids. The absolute scattering of polymer solutions was determined from transmission measurements and from two different transverse measurements. The experimental results are in good agreement. The variables of the photometric system were also analyzed and experimentally studied to determine its ability to measure absolute scattering of liquids under different geometrical arrangements.

## 1. Introduction

For the determination of the molecular weight of a solute by light scattering, the proportion of incident light that is dissipated as molecular scattering must be measured. This scattered light may be determined in either of two ways. First, the amount of light scattered at a given angle with respect to the incident beam of light may be experimentally determined when a given volume of illuminated solution is seen by a detector that has a well-defined angular acceptance. This method obviously requires that either a great number of dimensional details and optical constants be known or a comparison standard be available. Second, the amount of light scattered over all angles may be determined. The total scattered light may be determined by using an integrating sphere or by measuring the loss of light that occurs in the passage of a light beam through a given path of solution. The latter method of measuring total scattering requires that very precise transmission measurements be made, that extreme care be employed to eliminate secondary scattering in the experimental setup, and that materials be chosen that remove energy from the light beam only by molecular scattering and not by absorption or fluorescence. A molecular theory of light scattering is needed to relate the first experimental method, which measures the light scattered from a solution at a given angle, to the second method, which measures the total amount of light scattered at all angles.

The theories of Rayleigh [1, 2,]^1^ which apply to small isotropic particles, state that the intensity of scattering is proportional to the quantity (1+cos^2^ *θ*) when the incident light is unpolarized and *θ* is the angle measured from the direction of the incident beam to the direction of observation. The radiant intensity of scattered light, *J_θ_*, in the direction *θ*, from a volume, *V*, that is illuminated by an incident beam of irradiance *H*_0_, can be described by a parameter called Rayleigh’s ratio, *R_θ_*, as shown in eq (1).

$$R_{\theta }=\left(J_{\theta }/H_{0}V\right).$$ (1)

If now the scattering is summed up over all angles, the fractional decrease in the flux of the incident light for a small length, *dl*, of solution can be calculated and is known as the turbidity, *τ*. It is usually expressed in the integrated form shown in eq (2). In those cases where the above angular function (1 + cos^2^ *θ*) applies, the integration of the scattering over all angles yields a simple relation between Rayleigh’s ratio and the turbidity as shown in eq (3).

$$\tau =\frac{1}{l}\ln\frac{J}{J_{0}}$$ (2)

$$\tau =\left(16\pi /3\right)R_{90}.$$ (3)

By measuring both Rayleigh’s ratio at 90° and turbidity of a solution of isotropic particles, a light scattering photometer can be tested for self-consistency solely on the basis of optical determinations.

The work of Carr and Zimm [3] on the absolute scattering of liquids is based upon this approach. Doty and Steiner [4], in a study aimed at using spectrophotometric techniques for determining molecular weights, made a similar though less complete comparison. In spite of these efforts there still is an uncertainty about the absolute values of scattering from pure liquids [5]. The present work does not attempt to answer this molecular problem of liquids because it is not necessary for the ultimate use of this absolute photometer in molecular weight determinations. This work attempts to assess the reliability of the light scattering photometer, described earlier [6] for the determination of absolute scattering from solutions by comparing results from various optical measurements.

In addition to the direct optical methods of checking the consistency of a light scattering photometer, there are also indirect ways which use molecular theories to relate either the scattering of pure liquids to Avogadro’s number, or the scattering of solutions to the known molecular weight of the solute. Both of these indirect measurements have been carried out with this photometer although the results are not given at this time because greater care must be given to the preparation of “pure” liquids. A comparison of the molecular weights of polystyrene fractions determined by equilibrium ultracentrifugation and by light scattering will be published later.

## 2. Photometric Analysis

### 2.1. General Discussion

#### a. Definitions

If a source of radiant energy is designated 1, and a point in space from which the flux is measured is designated 2, then the following photometric quantities may be defined in terms of the area *A*, solid angle *d*Ω, and the angle *α* between the normal to the surface and the direction of irradiation:

radiant flux, *P*
radiant intensity, *J=dP/d*Ω, flux per unit solid angle.
radiance, *N=dJ/dA* cos *α*, intensity of the area *dA* projected perpendicularly to the direction *θ.*
irradiance, *H=dP/dA*_2_, flux incident on the small area *dA*_2_.
radiant emittance, *W=dP/dA*_1_, flux from the element of the surface *dA*_1_.

The incident irradiance from the lamp to a secondary source is referred to as *H*_0_. All other quantities without subscripts refer to the secondary source. Unfortunately two secondary sources need to be considered, a diffuser and a scattering solution, and accepted convention makes it necessary to talk about the directions from the source to the detector in different ways. For a plane diffuser the angle *θ* is measured from the normal to the surface of the diffuser. For a scattering solution the angle *θ* is measured from the forward direction of the beam.

#### b. Diffuser

Diffusing plates of magnesium oxide or magnesium carbonate are used frequently as attenuators in light scattering calibrations to provide a known fraction of the incident radiant flux. Since only the total diffuse reflectance has been determined for these diffusers, it is necessary to know that the particular diffuser used in the light scattering experiments redistributes the flux so that the radiance is equal in all directions. If this is true, it follows that the radiant intensity from the diffuser obeys Lambert’s law (as stated in eq (4)) and that the radiant emittance may be described in terms of the radiance by eq (4a).

$$J_{\theta }=J_{0}\cos\theta$$ (4)

W=πN. (4 a)

In order to relate the radiance of the diffuser to the incident irradiance additional experiments are necessary. Several workers [7, 8, 9] have shown that magnesium oxide and magnesium carbonate are not perfect diffusers because they do not diffusedly scatter all of the incident radiant flux. However, they are almost perfect in the visible region where they have a reflectance very close to unity. The incident irradiance, *H*_0_, on an ideal diffuser but imperfect reflector may be expressed as

$$r_{f}H_{0}\cos\;\alpha =\pi N,$$ (5)

where *α* is the angle between the direction of the incident beam and the normal to the diffuser surface and *r_f_* is the reflection factor.

#### c. Photometric Detection

The radiation detectors commonly used in light scattering measurements are photomultiplier tubes. These detectors usually do not have uniform sensitivity over the photosensitive surface, and for this reason they will not respond properly to the irradiance from two different sources unless the geometry of viewing is identical for the two sources. In any determination of absolute scattering where measurement of the ratio of irradiances from two different types of secondary sources, it is necessary to ensure that the flux density is uniform over the irradiated area.

#### d. Rayleigh's Ratio

The scattering function, termed Rayleigh’s ratio, is easily defined in terms of the small volume of scattering material, *V*, the distance of the detector from the scatterer, *r*, and the photometric quantities. Thus Rayleigh’s ratio may be written in the form of eq (1) or its equivalent forms in eq (1a).

$$R_{\theta }=\left(H_{\theta }r^{2}\right)/\left(H_{0}V\right)=N_{\theta }A_{1}\cos\theta /\left(H_{0}V\right).$$ (1a)

Since the scattering volume can easily be arranged to be equal to the product of the area of the field stop and the width of the incident light beam *w*, and since the same field stop can be used for the measurement of the incident irradiance, the ratio may also be written as in eq (6).

$$R_{\theta }=N_{\theta }\cos\;\theta /H_{0}w.$$ (6)

The Rayleigh’s ratio for a given isotropic material is generally considered to be the value at 90° to the incident beam where the cosine term becomes unity. Therefore, only the ratios, *N_θ_/H*_0_*w*, needs to be determined. To make this measurement accurately, the detector should be irradiated with nearly equal flux density from both incident and scattered beams. This may be accomplished by attenuation by absorption with calibrated filters, called in this work the direct method, or by attenuation by redistribution of the incident flux with a diffuser, referred to as the diffuser method.

**Figure 1:**
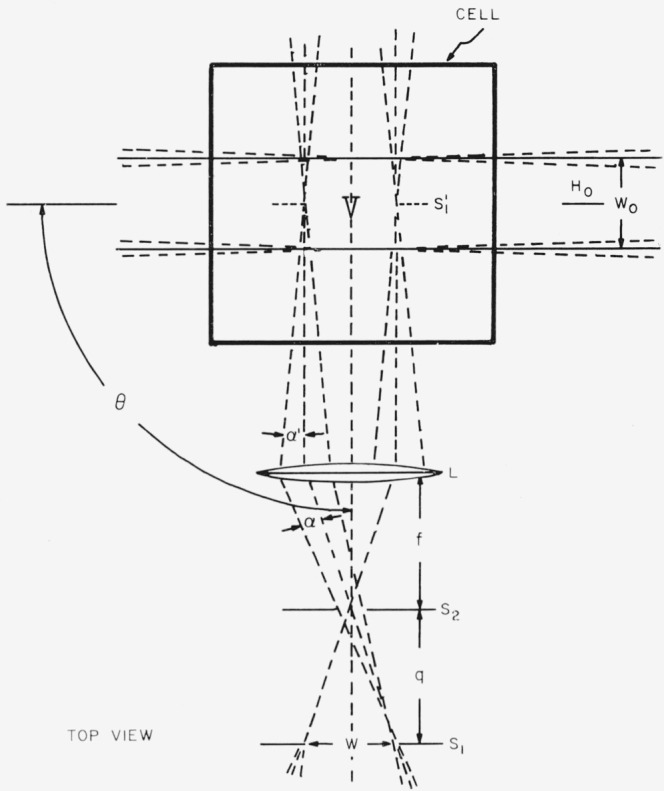
Detailed geometry of viewing for absolute scattering at right angles to the incident beam.

To visualize more easily the different dimensions that are associated with the determination of the absolute scattering, figure 1 is drawn with the detector viewing a scattering solution at 90°. The image *S*_1_′ of the field stop *S*_1_ at the center of the scattering volume has the dimensions *w*′*h*′. The incident beam of irradiance *H*_0_ has the area *w*_0_, *h*_0_, and thus defines a scattering volume for the detector of *w*_0_ *w*′*h*′.

### 2.2. Diffuser Method

In this case, the diffuser is substituted for the scattering volume shown in figure 1 so that the detector can measure the irradiance from source *H*_0_. Equation (5) may be substituted into eq (la) when the detector is viewing the diffuser at 90° to give eq (7).

$$R_{90}=\left(N_{90}/N_{\text{diff}}\right)\left(r_{f}\cos\;\alpha /\pi \right)\left(A_{\text{diff}}/V\right).$$ (7)

In this method only the radiance of the scatterei and diffuser need to be measured.

Since the area of the field stop that is used for the diffuser measurements is also known, Rayleigh’s ratio may be written as in eq (8).

$$R_{90}=\left(r_{f}\cos\;\alpha /\pi \right)\left(J_{90}/J_{\text{diff}}\right)\left(A_{\text{diff}}/V\right).$$ (8)

In the special case where the radiation incident on the diffuser has a projected area equal to the field stop in the diffuser measurement, Rayleigh’s ratio can very simply be written as eq (8a).

$$R_{90}=\left(r_{f}\cos\;\alpha /\pi \right)\left(J_{90}/J_{\text{diff}}\right)\left(1/w\right).$$ (8a)

For this case, the width of the field stop is the only geometrical factor that needs to be evaluated.

This experimental arrangement has been used frequently because the quantity *w*′ is the only dimensional measurement needed, and it can easily be determined by placing a real stop just in front of the scattering volume. However, two additional factors must then be examined. If the field stop is changed between the measurement of the diffuser and scatterer, then a simple relation between the radiant intensities and phototube responses may not be valid. Even if the limiting area in front of the phototube is maintained by a second lens that magnifies the image of the scatterer or diffuser, it must be known that the aperture stop of the second system is not changing simultaneously. Otherwise, the solid angle of acceptance changes and does not allow eq (8a) to be used without modification.

It seems preferable, therefore, to determine the uniformity of the light beam and then to sample a portion of the beam with the same field stop that is used in the 90° scattering measurement. In this way eq (8b) is used to calculate the Rayleigh’s ratio. The width of the incident beam is the only dimensional measurement that must be made, and this measurement can easily be made.

$$R_{90}=\left(r_{f}\cos\;\alpha /\pi \right)\left(N_{90}/N_{\text{diff}}\right)\left(\frac{1}{\omega_{0}}\right).$$ (8b)

### 2.3. Direct Method

#### a. Flux Through Two Stops

To evaluate Rayleigh’s ratio directly the radiant intensity of the light scattered from the solution must be determined so that eq (1) may be used. There have been two suggestions for the use of this method [10, 11]. One of these is a particularized solution of the other. Basically, one must calculate the flux passing through two apertures from a source that is located at one of the apertures. This is a very difficult problem to solve generally since it involves numerical approximations. However, the general outline of the problem can easily be seen, and the point at which approximations are introduced is easily recognized and the approximations evaluated.

**Figure 2:**
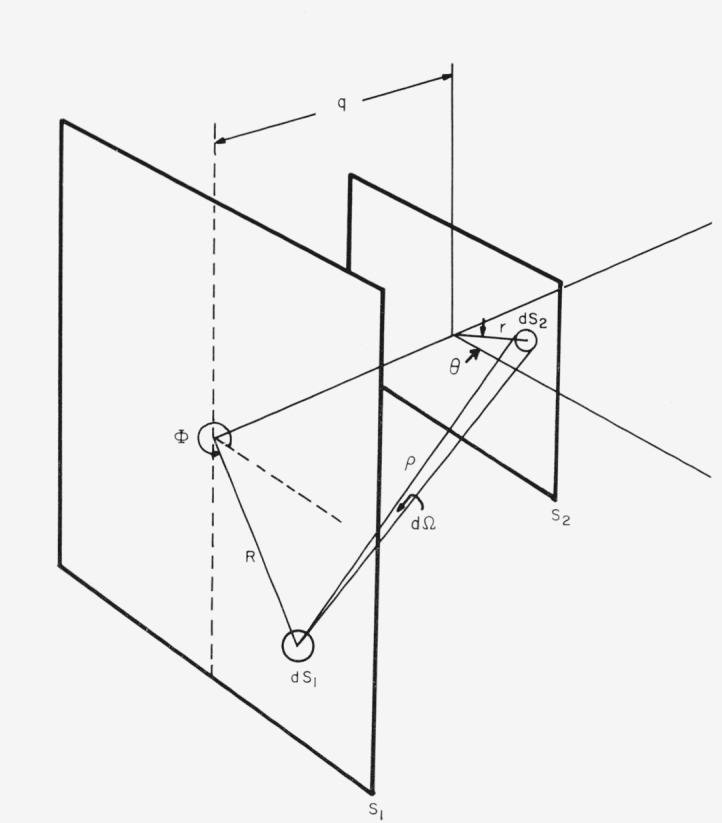
Diagram for the calculation of the flux passing through two stops.

If a lens is used, the stops either in image space or object space can be analyzed. Consider the system shown in figure 2, where *S*_1_ and *S*_2_ are parallel apertures (sometimes called stops) that are separated by a distance *q*, and the radiant source is located in the plane of one of the apertures *S*_1_ and has a radiance *N_θ_* in the direction of *θ.* The source is uniform over *S*_1_ so that the radiance is constant over the area. The solid angle, *d*Ω, from which the radiant flux is received at the second aperture is small enough so that the variation of the irradiance from the scattered light can be considered to be an average value equal to that at *θ.* Then the increment of radiant flux, *dP*, coming to the area *dS*_2_ on aperture *S*_2_, at a distance *ρ* from a small area *dS*_1_ on aperture *S*_1_, may be integrated to determine the total flux incident on *S*_2_.

$$\left(P\right)\text{at}\;dS_{2},\text{from}\;dS_{1}=N_{\theta }dS_{1}\left(d\Omega \right)=J_{\theta }d\Omega =J_{0}q\cdot \frac{dS_{2}}{\rho^{3}}$$ (9)

$$P=\int_{S_{1}}\int_{S_{2}}\left(J_{0}q\right)\frac{dS_{2}dS_{1}}{\rho^{3}}.$$ (10)

These equations lead to elliptic integrals and numerical integration for any stop system, circular or rectangular. However, an approximation may be easily made in circular coordinates that will be valid for circular stop systems. Equation (10) can then be written as eq (11).

$$P=J_{\theta }\int_{S_{1}}dS_{1}\int_{S_{2}}\frac{qrdrd\theta }{{\left[R^{2}+r^{2}+2Rr\cos\left(\theta -\Phi \right)+q^{2}\right]}^{3/2}}.$$ (11)

If the aperture stop in the optical system is small compared to the interstop distance and the field stop, then the above expression may be integrated and expanded in powers of the ratio of field stop radius to interstop distance as shown in eq (12).

$$P=J_{90}\left(S_{1}S_{2}/q^{2}\right)\left[1-3/4{\left(r/q\right)}^{2}+\ldots \right].$$ (12)

For the setup used in these experiments, the approximation in eq (12) involves neglecting a term contributing less than ⅓ percent to the series before the first integration. Thus the use of the term (*S*_1_*S*_2_*/q*^2^) *J*_90_ to measure the flux would indicate an error less than 1 percent from the true value.

*R*_90_ can be evaluated from eqs (12) and (1) by measuring the stop geometry, the irradiated volume and *H*_0_. A special case of this general photometric analysis is the telecentric optical system. The irradiance from the source is accepted in such a way that the principal ray always passes through the principal point of the lens because the aperture stop is centered at this position. If the focal length of the lens is *f*, then the magnification, *m*, of the system according to Newton’s formula is *f*/*q*, so that the viewed area will be *S*_1_(*m*)^2^. In this case the flux can be represented as shown in eq (13).

$$P=J_{90}\left(S_{1}S_{2}/f^{2}\right).$$ (13)

Only the aperture stop and focal length must be known to determine the radiant intensity in such an optical system.

A scattering solution, however, occupies volume and may not be exactly centered in the plane of one of the stops. In this case the scattering surface can be considered to be moved toward or away from stop *S*_2_. The angular acceptance of the system increases in the same proportion that the area of the source is decreased by the aperture stop provided that the movement of the surface is much less than the interstop distance.

#### b. Volume Correction

The total volume contributing to the scattering is not that defined by a principal ray but rather the volume that is defined by a pencil of rays over the entire aperture stop. If the ray that passes through the center of the aperture stop also passes through the focal plane, there is no volume correction because the zones in the field of view for which only a portion of the aperture is filled are equally compensated by those portions of the field that pass symmetrically on the other side of the aperture stop. If, on the other hand, the ray through the center of the aperture stop is not also the ray through the focal point, the scattering volume will be that defined by the rays through the center of the aperture stop.

#### c. Refraction Correction

Hermans and Levinson [12] have determined the effect of viewing a radiant source through a system of two stops when the source is located in a medium of refractive index *n.* They found that in all cases where the image of the field stop did not exceed the dimensions of the radiant source, the angular acceptance would be proportional to *n*^2^. Their calculation was based upon an optical system that had the same dimensional restrictions as ours, but had the additional implicit restriction that the stops be much smaller in a linear dimension than the interstop distance.

#### d. Reflection Correction

The Fresnel formula for computing reflection at normal incidence allows a correction to be made easily for reflections from the glass-air interfaces in these experiments. The correction needs to be made only when the direct beam is being measured through a square cell. In this case, the additional scattering due to reflection of the scattered beam from the back surface of the cell and the reflection of the incident beam where it leaves the cell is subtracted from the 90° measurement of scattering. When the incident beam irradiance is measured without the cell in place, the loss of light in entering the cell and leaving the cell is just compensated by the back reflections discussed above. The Fresnel reflections from the liquids to the glass, when they are not isorefractive, are usually no more than 0.2 percent. For soft glass cells, the refractive index for the sodium *D* line is 1.516, and the calculated indexes for 436 m*μ* and 546 m*μ* are 1.524 and 1.517, respectively. The 90° light scattering measurements are thus decreased by factors of 0.915 and 0.913, respectively. For a Pyrex cell the reflectance is computed to increase by about a percent to 0.927 at 546 m*μ* if the refractive index of the Pyrex is 1.474.

## 3. Experimental Measurements

### 3.1 Materials

#### a. Cells

Several different types of light scattering cells were used. Two of them were 44 mm semi-octagonal cells. One cell was coated with a black absorbing paint on the back; the other was uncoated. Two other cells had cross sections 37 mm square. One was made of Pyrex glass; the other of lime glass. A fifth rectangular cell was used that could be tightly sealed. A seventh cell was a specially constructed cell made of Pyrex glass, measuring 55 × 10 × 10 mm, and had extreme close tolerances on angles and high clarity in the fused joints. A sixth cell was a Rayleigh horn which had 1-in. fused Pyrex circular entrance and exit windows and a total volume of 200 ml.

#### b. Solutions

The polystyrene samples used in these measurements were fractions that had been prepared for work on molecular weight determinations. The fraction used for the comparison of transverse scattering and transmission measurement had a molecular weight of 390,000. The fraction used for studies of the variables involved in low-level light scattering from solutions had a molecular weight of 150,000. Another fraction of 50,000 molecular weight was also studied. The strongly scattering solutions of colloidal silica were selected samples of Ludox.^2^ Attempts to determine aggregation by the dissymmetry of the scattered light using four Ludox samples on hand showed little difference.

Reagent-grade cyclohexane was used. After a fractional distillation from a glass helix-packed column it had a refractive index $n_{D}^{30}$ of 1.4205. Methyl ethyl ketone (Matheson) was also prepared by distillation from a glass helix-packed column; it had a refractive index $n_{D}^{30}$ of 1.3738. Benzene and toluene were prepared from reagent grade (ACS) materials by fractionally distilling from a pot containing sodium. A spectroscopic-grade sample of carbon tetrachloride was measured without purification, then after a simple distillation, and again after a treatment with sodium hydroxide followed by drying and distillation.

All solutions of polystyrene in cyclohexane were handled in a large heated box.

#### c. Diffusers

Magnesium oxide diffusers were prepared in two ways. Magnesium oxide was obtained on an aluminum plaque 2 in. in diameter by burning magnesium turnings [13]. On several occasions a magnesium oxide cake was prepared from ACS reagent-grade magnesium oxide powder by packing it into a brass holder and then pressing it together lightly with a piece of glass.

Magnesium carbonate diffusers were surfaced by drawing a straight edge over the surface of magnesia blocks. In addition, a piece of white Vitrolite [14] was used as a reference diffuser after calibration with magnesium oxide in the light scattering photometer.

Table 1 shows the relative intensity as a function of viewing angle for a magnesium oxide diffusing surface irradiated by a 1 mm beam of light of wavelength 546 m*μ* incident at 45°.

The divergence of the incident beam was less than 0.1° in all directions and the angular acceptance of the receiver was 2.5°. Column 4 lists the function cos *α* which corresponds to the relative irradiance from a Lambertian diffuser. Column 5 lists the polarization ratio for the smoked oxide that is, the ratio of horizontally to vertically polarized scattered light. In view of the relatively good agreement of cols. 2 and 3 with col. 4, the diffusers were considered to obey Lambert’s law to a satisfactory degree.

Diffusers are illuminated by a relatively large beam, usually of the order of 10 mm in width, when they are used in light scattering calibration. Table 2 shows the results obtained with the above diffusers when they had a 9.9×12 mm rectangular light beam of wavelength 546 m*μ* incident upon them at 45°. The angular acceptance of the receiver was again 2.5°, and the field of view was 6×11 mm at all times. In this setup, measurements beyond 80° must be excluded since the projected area of the receiver field of view becomes larger than the width of the beam. The values have also been obtained for 436 m*μ* and are similar. Magnesium carbonate was studied in the same manner and the results are shown in table 3 for both the large and small beams.

Table 4 shows the ratios obtained by direct comparison of the intensities from MgO and MgCO_3_ as well as for Vitrolite and MgO under conditions of 45° incidence and normal viewing when using freshly smoked MgO and freshly scraped MgCO_3_. In order to obtain more information about possible variations of these diffusers, a pressed MgO and a smoked MgO were compared directly with the Vitrolite. The reflectance ratios at 546 m*μ* and 436 m*μ*, respectively, were 0.855 and 0.840 for the pressed sample, and 0.854 and 0.840 for the smoked.

Columns (5) and (6) of table 3 contain the radiance variations and polarization of another MgCO_3_ diffuser which was used to obtain a constant luminance over a larger angular range. Both samples were measured for total reflectance relative to freshly smoked MgO and found to be within 1 percent in the green portion of the spectrum but different by about 5 percent in the blue. A similar slight decrease in the total reflectance at 436 m*μ* of the magnesia blocks was found later. The magnesium oxide did not show any appreciable reflectance changes with time.

The total reflectance of the magnesium oxide diffuser was determined several times under the same viewing conditions and also, as will be discussed later, several times under different viewing conditions. The total reflectance was determined from eq (5) in which the incident irradiance *H*_0_ was determined by reference to neutral filters calibrated at the same time. The reflectances of Vitrolite, which does not have a fragile surface and can be assumed to have a greater permanence, were also determined at the same time. The measured Vitrolite reflectances were repeatable within 1.3 percent in the green and 0.8 percent in the blue over the course of several months. The MgO over the same period varied by 1.6 percent in the green and 1.3 percent in the blue. The reflection factor of the MgO diffuser was determined to be 0.967 in the green and 0.888 in the blue.

### 3.2. Transmission Measurements

Transmission measurements were made by using 5 cm or 10 cm Beckman cells. The transmission cell holder was built with a jacket so that water from a constant-temperature bath could circulate to keep constant temperatures in the cell at temperatures not too far from ambient temperature. The blackened thermostat was slightly longer than the cell to minimize the effect of ambient air on the cell ends. The experimental setup was very adaptable, and in view of Heller and Tabibian’s work [15] it was decided to investigate any effects of secondary scattering photographically as well as photoelectrically. With a 2 mm incident beam, having an angular divergence of 0.3°, no difference could be detected in the photographs when a solution of 3 percent Ludox or a sample of ultracentrifuged water was inserted into the transmission cell and placed on the table. The camera was focused on the center of the cell with the aperture wide open so as not to fail to detect any secondary scattering. A stray-light baffle did seem to help somewhat if it was placed about 4 in. on the source side of the cell center.

Measurements were made at 546 m*μ* for different dilutions of a high turbidity (0.123 cm^−1^) Ludox solution in a 10 cm cell using a 2 mm beam of 0.07° divergence and a receiver allowing use of different fields of view and angular acceptance. With a 5.5×9.9 mm field of view and angular acceptances of both 0.5° and 4° the measured turbidity increased less than 2 percent. With a 2 mm diameter circular field of view and the same angular acceptances there was no change in the transmission measurements within experimental error.

The small amount of secondary scattering is unquestionably due to the very small size and angular divergence of the light beam used in these transmission measurements. The turbidity was obtained from transmission measurements using a 4 mm diameter circular field of view. Equation (2) was used to calculate the turbidity where *J*_0_ and *J* are the radiant intensities of the incident and transmitted beams, respectively, after subtraction of any solvent absorption blank, and *l* is the solution path length in cm. The turbidity can be related to the transversely scattered light by eq (2) only in special cases. In general, a particle scattering factor is required, and it is different in transmission and transverse measurements.

### 3.3. Transverse Measurements

Since a wide variety of stop arrangements were available in the instrument, several experiments were conducted to test the applicability of eq (12) relating the radiant flux to the stop areas. In addition, tests were made to ascertain what effects the beam divergence and the volume of irradiated solution had on the measurement of the flux from the scattered light. Equation (12) states that if the radius of the receiver stop is less than 110 of the interstop distance the radiant flux received will be directly proportional to the product of the stop areas to within 1 percent. With a fixed interstop distance and a fixed field stop in front of the phototube the ratio of the aperture stop area (proportional to diameter squared, *D*^2^) to the phototube signal, *G*, should be a constant. Table 5 reports ratios of *D*^2^*/G* obtained with two solutions of vastly different scattering intensities (polystyrene and Ludox) and also from a magnesium oxide diffuser. The low-molecular weight polystyrene fraction in cyclohexane had a turbidity of approximately 0.008 cm^−1^ while the Ludox solution had a turbidity of approximately 0.07 cm^−1^. Table 5 lists the diameter of the aperture stop in col. (1) and its half angle in col. (2), that is, half of the conical apex angle defined by the circular aperture stop. The footnotes describe the size of the incident beam, its half angle of divergence, and also the size of the field stop. The linear dimensions of the image of the field stop seen by the phototube are magnified 1.1 times.

The effect of the divergence of the incident beam on the scattering measurement from these weakly scattering solutions is shown to be quite small, though not negligible, in table 6.

The analysis of this optical system indicated that the volume of scattering should be calculable from the field stop image and the width of the incident beam. To see if edge effects were actually important, a comparison was made on the above polystyrene solution with a 3 mm and also a 9 mm incident beam whose dimensions had been measured earlier [6]. At 546 m*μ* the ratio of the scattered flux intensities when using these two beam widths was 2.93 while the ratio calculated on the basis of the measured widths was 2.95.

Another conclusion of the previous photometric discussion is that the position of the field stop image with respect to the exact center of the secondary scattering source should not be critical in defining the scattering volume. Table 7 reports the experimental measurements on the polystyrene solution as well as on the magnesium oxide diffuser. Column 1 lists the distance of the field stop image from the center of the scattering, col. 2 lists the data for the polystyrene solution, and cols. 3 and 4 list the data for the magnesium oxide diffuser for two different acceptance angles, 2.9° and 1.8°, respectively. The above experiments examine the effects of not locating the image exactly in the center of the field stop and show very little difference.

The effects of the volume correction were also examined to determine if the edge effects in the volume had really been eliminated by assuming that the principal ray in this telecentric system did define the volume that was to be considered in Rayleigh’s ratio. For this purpose several field stops were used to measure the scattering from the same solution irradiated by the same beam. The ratio of scattered to incident radiation was then determined. Table 8 shows that a doubling of the scattering volume has little effect on the measured scattering.

The scattering is almost constant. Since the results appear random, and since the incident beam is known to be uniform over its area [6], it appears that the scattering volume has been properly treated.

Another possible source of error in transverse measurements arises from a cell construction that allows stray light to interfere with low-level scattering measurements. An attempt was made to determine if cell construction had very much effect upon the determination of scattering from pure solvents like benzene and cyclohexane. Extraneous scattering from the cell walls should be most noticeable at these low levels of scattering. Although there is no easy way to explore the errors in cell design systematically, several quite different cells were examined with the same solvent to observe any large changes in scattering values. Table 9 shows the results obtained using benzene in the various cells described earlier, for both 546 m*μ* and 436 m*μ* light. The projected incident beam was 10 mm wide; it had an angular divergence of 0.5° in the horizontal direction and 1° in the vertical direction. The receiver accepted a 2.5° half angle and viewed an area 6×11 mm. The benzene was thermostatted at 25° C. The scattering from the Rayleigh horns were corrected for the loss of the back reflections by increasing the scattering ratio for the Fresnel reflections at two Pyrex surfaces. The ratio for the semioctagonal cell that had been coated on its back surface with an absorbing paint was increased by the lime-glass correction for one reflection surface. The data given are for the ratio of the scattered beam signal to the incident beam signal. These different cells do not give as widely different values of absolute scattering as might have been assumed; however, the depolarization values vary considerably. In the case of carbon tetrachloride successive purification was necessary to reduce the depolarization apparently due to some fluorescing impurity.

### 3.4. Comparative Transverse and Transmission Measurements

#### a. Polystyrene Solutions

Experiments were made on solutions of polystyrene to determine Rayleigh’s ratio by transmission measurements as well as by two different transverse scattering measurements. The transverse measurements involved the use of eq (8b) when the diffuser was used, and eq (13) when the stop theory was used. In general, the transmission turbidity was determined by eq (2) only for solutions which scattered so strongly that extremely accurate transmission turbidities, better than the 0.2–0.3 percent available, were not needed. In one case, the scattering from the same solution was measured by two different types of photomultipliers, the 1P21 and the end-on type 5819 in order to ascertain the presence of any small differences due to the overshooting of the photosensitive surface by the incoming rays.

The solutions were cleaned by filtration through ultrafine filters and were measured in a semioctagonal cell so that the high-angle dissymmetry of scattering was always easy to establish. In some cases the solutions were first used in the semioctagonal cells, then put into the transmission cell, and again introduced into the semi-octagonal cell to see if time and repeated handling perceptibly changed any of the scattering values. The values of the scattering always stayed the same within 0.5 percent. The results are listed in table 10. The 45°/135° dissymmetry of scattering for these strongly turbid solutions made up from the 390,000 molecular weight polystyrene fraction was about 1.1. The fraction used in this work was examined closely for depolarization and fluorescence effects. The depolarization ratio at 90° was always less than 0.01 and the fluorescence was less than 0.3 percent when it was present.

#### b. Ludox

A stock solution of Ludox about two years old was examined in order to check the consistency of the transmission turbidity and transverse scattering from water media at very high turbidities. It was realized that, in view of Goring’s work [16], such comparisons might be in error because of the discrepancy which can occur between the transmission turbidity and the integrated transverse scattering when there is light absorption by the Ludox. The water used to dilute the Ludox was clarified by ultracentrifugation, and the Ludox was filtered through sintered glass filters. Solutions of 1, 2, 3, and 4 percent were prepared from a filtered stock solution of 6 percent. The angular dissymmetry (45°/135°) measurements throughout the experiments were about 1.05. This is not the ultimate cleanliness of the samples, but merely the level which allowed quick and easy filtration through fine sintered glass filters. At this level the scattering factor is low enough to make the corrections to both the transverse and transmission measurements negligible for the purposes of these measurements.

Since many people have used this particular technique to calibrate their photometers, it was of interest to us to find out how routine use of this method would agree with our other determinations of scattering. The experimental measurement of *R*_90_ and *τ* was similar to that described earlier for the polystyrene solutions. The ratio (16*π*/3) *R*_90_/*τ* was plotted against concentration to give a curve which on extrapolation to zero concentration gave a value of 0.98 for light at 436 m*μ* and 0.97 at 546 m*μ*. Theoretically the intercept should be 1.000. The reason for this difference was not investigated further but could be due to a small amount of absorption by the solute. The slopes of the curves do give some measure of the amount of secondary scattering which, as is to be expected, is smaller for the 546 m*μ* light than the 436 m*μ*.

The increased secondary scattering at higher concentrations also causes the depolarization ratios to change from 0.033 and 0.012 for a 4 percent solution at 436 m*μ* and 546 m*μ*, respectively, to 0.008 and 0.008 at 0.5 percent.

## 4. Discussion

### 4.1. Agreement of the Different Scattering Methods

The data in table 10 demonstrate the very good agreement of the transverse scattering determined by the use of the diffuse reflector and the direct measurement of the incident beam intensity. These two scattering measurements are different from their respective forms in eqs (8) and (13) only in that a reflectance factor for the cell is calculated from the Fresnel equation and applied to the direct-beam measurement. The results for the 546 m*μ* wavelength light are consistent and make these transverse scattering measurements extremely reliable. However, a disturbing fact arises for the results at 436 m*μ* because the direct-beam measurements and diffuser measurements agree within a few percent even though the experimental determination of the absolute reflectance of the magnesium oxide is 0.88 rather than the literature value of 0.96 which was used to calculate *R*_90_.

The agreement of the transmission turbidities and the transverse scattering are within ±1.5 percent in all but one case. However, this particular run must have been in error since the results of the direct transverse measurement and the diffuser transverse measurement are also different. In both green and blue light, the agreement of the turbidities is equally good. It must be emphasized at this point that only impurities in the polymer sample could have an effect on the transmission since the solution transmission was always referred to the solvent. The transverse scattering measurements which were made in semioctagonal cells are correct, even if the sample absorbs light, because the incident beam is measured after passage through the cell. The additional results with lower molecular weight samples indicate that even at very low turbidities, where the transmission results are subject to great errors, the two methods of transverse scattering agree. It should also be pointed out, as the data in table 10 indicate, that two different solvents were used. The turbidity level was correspondingly decreased at the same concentration so that any absorption effects would be expected to become more predominant. These data indicate that such an effect is not present. Since depolarization and fluorescence were shown to be small, the equating of the transmission turbidity to the transverse scattering by the simple eq (3) is valid. Any corrections due to the different Cabannes’ depolarization factor for transverse scattering and integrated scattering would be insignificant.

The results of the Ludox experiments indicate that any calibration with such a highly scattering substance is bound to be difficult because of secondary scattering most pronounced in blue light. However, the agreement of the Ludox results with the polystyrene calibrations indicates that the refractive index correction for the square cell is correct for the small refractive index range 1.33 to 1.42.

### 4.2. Transverse Scattering Measurements

As the data in table 5 indicate, the flux is a linear function of the area of the stop for a half-angle acceptance range of 4° to less than 1°. The value for the half-angle of 0.55° is included even though it is not in line with the other results. Inspection of the 0.55° stop under a coordinate comparator showed that the hole had been drilled quite unevenly. The scattering from the isotropic polystyrene solution of low turbidity naturally gives the best results in this test of the instrument over its range of angular acceptance. The measurements of the angular acceptance using magnesium oxide as a scatterer are not as consistent as the results with scattering solutions.

The results indicate that the freshly prepared diffusers agree very well in relation to each other and with respect to the relative literature values for MgO and MgCO_3_. The experimental value for the particular sample of Vitrolite agreed with that determined with an integrating sphere. Furthermore the agreement with Lambert’s law was satisfactory in all of the samples used. It should be noted that making a MgO diffuser by packing MgO powder in a plaque did not at first yield good results, but gradually a technique was developed in which a Lambertian diffuser could be prepared at will.

The very good agreement mentioned earlier when comparing two different beam widths indicates that the calculations which predict no volume effects in scattering when using this type of receiver are correct. Similarly, the data in table 7 indicate that the calculation is correct in its prediction that the scattered flux is unchanged if the stop is imaged behind or in front of the center of the source. The results in table 8 also indicate that even when the field of view is halved the scattering ratio remains the same so that the volume correction is adequate.

The results with pure solvents in different types of cells is highly encouraging because it suggests that even at low turbidities the cells are not contributing stray light. The data in table 9 suggest that for our instrument the measured solvent scattering is not affected appreciably by cell design, although such effects can never be said to be absolutely eliminated. However, the high scattering value for benzene at 436 m*μ* in the semi-octagonal cell illustrates some of the dangers and problems in making low-level measurements. It also points out the value of making additional measurements of depolarization and fluorescence, especially at 436 m*μ.* At first, the apparent discrepancy in cells was difficult to understand. After many days of retesting and rinsing, it was discovered that this cell had been cleaned at one time with a detergent that had a strongly fluorescing material. Only after a lengthy treatment with strong acid did the scattering in this cell revert to the lower values obtained with the other cells. The values obtained at 546 m*μ* were at all times normal.

### 4.3. Transmission Measurements

The transmission values are very reproducible. The same values have been measured whatever variety of experimental arrangements has been used. At one time a diffuser was even inserted before the phototube to make certain that any possible geometrical changes in the positioning of the small beam used for transmission measurements were not producing erroneous results by allowing light to strike the photosensitive surface at different places. The results were the same with or without the diffuser.

## 5. Summary

A light-scattering photometer has been examined by two independent optical means, that is, by transmission and transverse scattering measurements, and the results shown to be in agreement. The results of this study indicate that the instrument is capable of a diversity of different arrangements which give reliable results. It should allow a more extensive investigation of the theory and practice of light scattering. The extension of these measurements to molecular weight determinations and other absolute liquid scattering should be a straightforward experimental procedure when clarification techniques become more reproducible.

## Figures and Tables

**Table 1:** Angular variation of relative intensity from various magnesium oxide diffusers irradiated by a 1 mm beam

(1)	(2)	(3)	(4)	(5)
*α*	Smoked MgO	Pressed MgO	COS *α*	Polarization ratio *ρ*u (Smoked MgO)
				
−15	0.969	0.983	0.966	0.987
−5	.998	1.000	.996	.961
0	1.000	1.000	1.000	.952
5	0.996	0.994	0.996	.932
15	.955	.971	.966	.903
25	.889	.913	.906	.883
35	.799	.830	.819	.859
45	.686	.719	.707	.822
55	.559	.580	.574	.818
65	.410	.425	.423	.799
75	.352	.253	.259	.791

**Table 2:** Angular variation of radiance for MgO using a 9.9 mm beam, normalized to unity for α=0

α	Smoked MgO	Pressed MgO	Polarization ratio *ρ*u (smoked MgO)
			
−15	1.000	1.006	0.992
−10		1.005	
−5	1.000	1.005	
0	1.000	1.000	
5	1.000	0.996	.945
10		.993	
15		.993	
20		.992	
25	1.000	.992	
30		.993	
35		.994	
40	1.002	.989	
45	1.010	1.003	.827
50		1.007	
55	1.022	1.014	.807
60		1.021	
65			
70		1.007	

**Table 3:** Angular variation of radiance for MgCO_3_ diffusers, normalized to unity for α=0

(1)	(2)	(3)	(4)	(5)	(6)
*α*	Diffuser 1			Diffuser 2	
	1 mm beam	10 mm beam	*ρ*u	10 mm beam	*ρ*u
					
−15	0.996	0.998		1.000	
−5	.997	.998			
0	1.000	1.000		1.000	0.966
5	1.002	1.001	0.869	0.999	
15	0.996	1.001		.995	
25	.998	1.008		1.000	
35	1.009	1.012		1.000	
45	1.006	1.028	.916	1.006	.877
55	1.002	1.047	.985	1.025	
65	0.998	1.073			
75	1.050	1.091	.957		

**Table 4:** Scattering ratios for different types of diffusers

Diffusing media	Scattering ratio for indicated wavelength	
		
	*546 mμ*	*436 mμ*
MgCO_3_/MgO	0.995	0.990
Vitrolite/MgO	.850	.838

**Table 5:** Ratio of aperture stop to phototube response for different viewing conditions

(1) Aperture stop	(2) Half angle	(3) aD^2^/G	(4) bD^2^/G	(5) cD^2^/G	(6) dD^2^/G	(7) eD^2^/G
						
*Diameter, D, mm*	*deqrees*					
4.08	2.9	2.91	2.80	5.55	1.39	1.69
3.150	2.2	2.90	……	……	……	……
2.479	1 8	2.84	2.87	……	1.40	1.71
1.320	0.936	2.93	……	5.51	1.44	……
0.975	.089	2.88	2.87	……	1.55	……
.772	.55	3.17	……	5.73	1.50	1.78

a Polystyrene solution: 10mm beam, 0.07° divergence; 5.5×9.9 mm field stop.

b Polystyrene solution: 10mm beam, 0.07° divergence; 5.04 mm diam. field stop.

c Ludox: 10 mm beam, 0.07° divergence; 5.04 mm diam. field stop.

d MgO: 10 mm beam, 0.07° divergence; 5.04 mm diam. field stop.

e MgO: 10 mm beam, approximately 0.3° divergence; 5.5×9.9 mm field stop.

**Table 6:** Effect of divergence of incident beam on the measured scattering

Wavelength	Scattering response	
	0.07° Div. vertical	3° Div. vertical
	0.07° Div. horizontal	1° Div. horizontal
		
546 m*μ*	1.124	1.137
436 m*μ*	1.033	1.004

**Table 7:** Effect of distance between field stop image and the scattering volume center

(1)	(2)	(3)	(4)
Distance	Polystyrene	MgO	
			
*cm*		*2.9*°	*1.8*°
0	59.5	85.2	41.0
8.1	59.8	85.0	41.2

**Table 8:** Effect of the field stop on the measured scattering

Field stop dimensions	Scattering	
		
*mm*	*436 mμ*	*546 mμ*
3.0×7.3	1.68	6.44
5.04 dia	1.82	6.64
5.5×9.9	1.74	6.54

**Table 9:** Scattering from benzene in different cell*s*

	37mm×37mm square		44mm semi-octagonal		5.5×10mm×10mm	
						
	Soft glass	Pyrex	Soft unpainted	Soft back painted	Pyrex	Horn Pyrex
	
	546 m*μ*					
	
Scattering	0.566	0.563	0.552	0.552	0.553	0.552
Depolarization	.398	.410	.405	.413	.405	.411
	
	436 m*μ*					
	
Scattering	1.450	……	1.480	……	1.436	1.448
Depolarization	0.443	……	0.400	……	0.420	0.429

**Table 10:** Comparison of transverse and transmission turbidities for polystyrene solutions

Turbidities (cm^−1^×10^3^)						
Polystyrene (400,000 MW)	Solvent	Temp.	Phototube	Transverse		Transmission
				Direct	Diffuser	

At 546 m*μ*						

*conc* (*g/dl*)						
0.94	cyclohexane	37°	1P21	14.3	14.4	14.4
			5819	14.2	14.3	14.0
1.10	do	37°	5819	15.44	15.2	15.08
0.90	butanone	room	5819	9.79	9.64	9.65

At 436 m*μ*						

0.94	cyclohexane.	37°	1P21	38.7	35.4	38.60
			5819	38.1	37.8	38.60
1.10	do	37°	5819	41.1	39.5	40.5
0.90	butanone	room	5819	25.2	24.8	26.5

## Footnotes

1 Figures in brackets indicate the literature references at the end of this paper.

2 Trademark of E. I. du Pont de Nemours & Co., Inc.
